# Supplementary material for: A key spectral tuning site of UV-sensitive vertebrate non-visual opsin Opn5
Source: Cell Mol Life Sci. 2025 Sep 2;82(1):334. doi: 10.1007/s00018-025-05879-3 (PMC12405127; doi:10.1007/s00018-025-05879-3)
Supplement: Supplementary file 1 — (DOCX 948 KB) [file 18_2025_5879_MOESM1_ESM.docx]

Supplementary information for

Title: A key spectral tuning site of UV-sensitive vertebrate non-visual opsin Opn5

Authors: Takahiro Yamashita ^1^*, Kazuyuki Asamoto ^1^, Kengo Fujii ^1^, Chihiro Fujiyabu ^1^, Hideyo Ohuchi ^2^, Yoshinori Shichida ^1,3^

Affiliations: ^1^ Department of Biophysics, Graduate School of Science, Kyoto University, Kyoto 606-8502, Japan; ^2^ Department of Cytology and Histology, Okayama University Graduate School of Medicine, Dentistry and Pharmaceutical Sciences, Okayama 700-8558, Japan; ^3^ Research Organization for Science and Technology, Ritsumeikan University, Shiga 525-8577, Japan

*Corresponding author: Takahiro Yamashita, Department of Biophysics, Graduate School of Science, Kyoto University, Kyoto 606-8502, Japan. Email: yamashita.takahiro.4z@kyoto-u.ac.jp

**
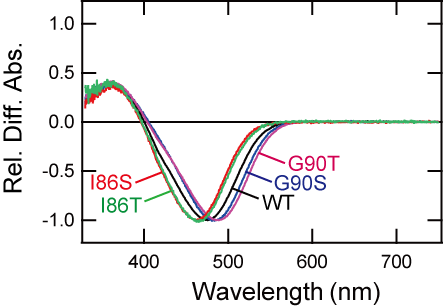
**

**Figure S1 Comparison of spectral property of I86 and G90 mutants**

Difference spectra shown as curve 1 in Fig. 2C-2G were normalized to be ~ -1.0 at the negative maximum.


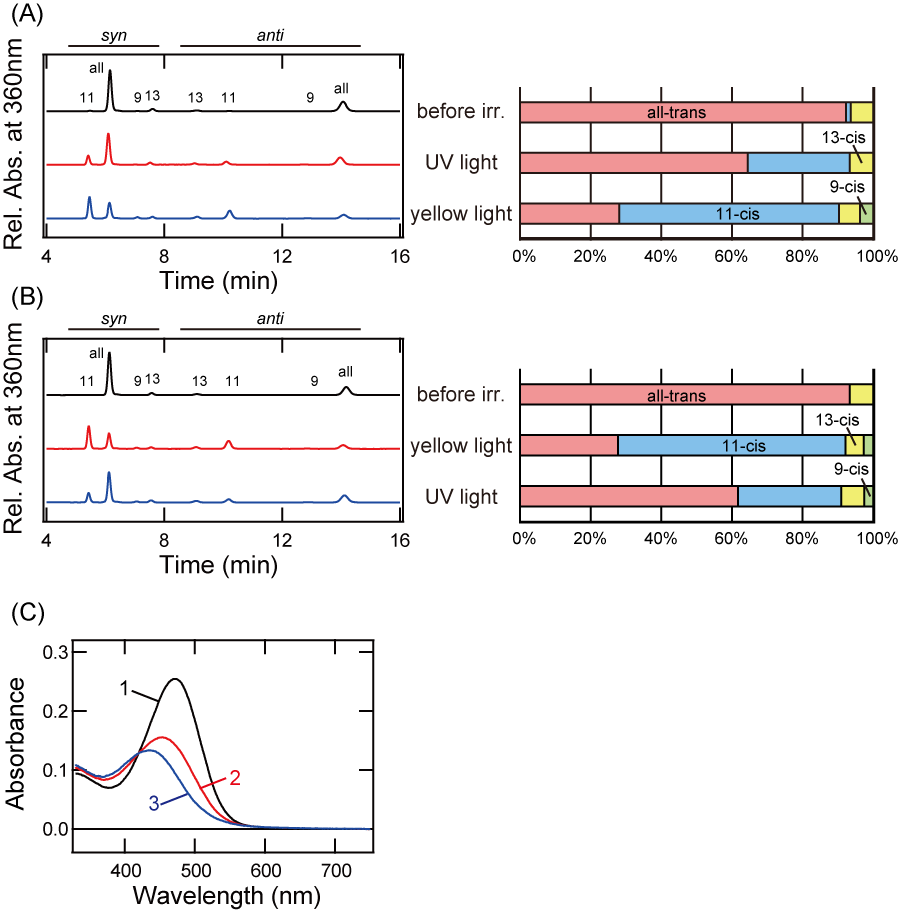


**Figure S2 Characterization of K91T mutant of Opn5m**

(A) Retinal configuration changes of K91T mutant purified after the incubation with 11-*cis* retinal. The configurations were analyzed before light irradiation, after UV light (360 nm) irradiation and after subsequent yellow light (>500 nm) irradiation. (B) Retinal configuration changes of K91T mutant purified after the incubation with all-*trans* retinal. The configurations were analyzed before light irradiation, after yellow light irradiation and after subsequent UV light irradiation. (Left) The retinal configurations were analyzed with HPLC after extraction of the chromophore as retinal oximes (syn and anti forms of 9-*cis*, 11-*cis*, 13-*cis*, and all-*trans* retinal oximes). (Right) Isomeric compositions of retinal before and after light irradiation of the mutant. (C) Calculation of absorption spectra of K91T mutant. We recorded the absorption spectrum (curve 1) of the sample purified after reconstitution with all-*trans* retinal (the same as curve 1 of Fig. 3B). This sample contained almost exclusively all-*trans* retinal (see Fig. S2B), which means that curve 1 corresponds to the spectrum of the all-*trans* retinal bound form. To obtain the absorption spectrum of the 11-*cis* retinal bound form (curve 3), we subtracted curve 1 from curve 2 (the same as curve 2 of Fig. 3B) based on the component ratio of 11-*cis* and all-*trans* retinals shown in Fig. S2B. Finally, we normalized curves 1 and 3 to be ~1.0 at λmax of curve 1 to show the normalized spectra in Fig. 3C.

**
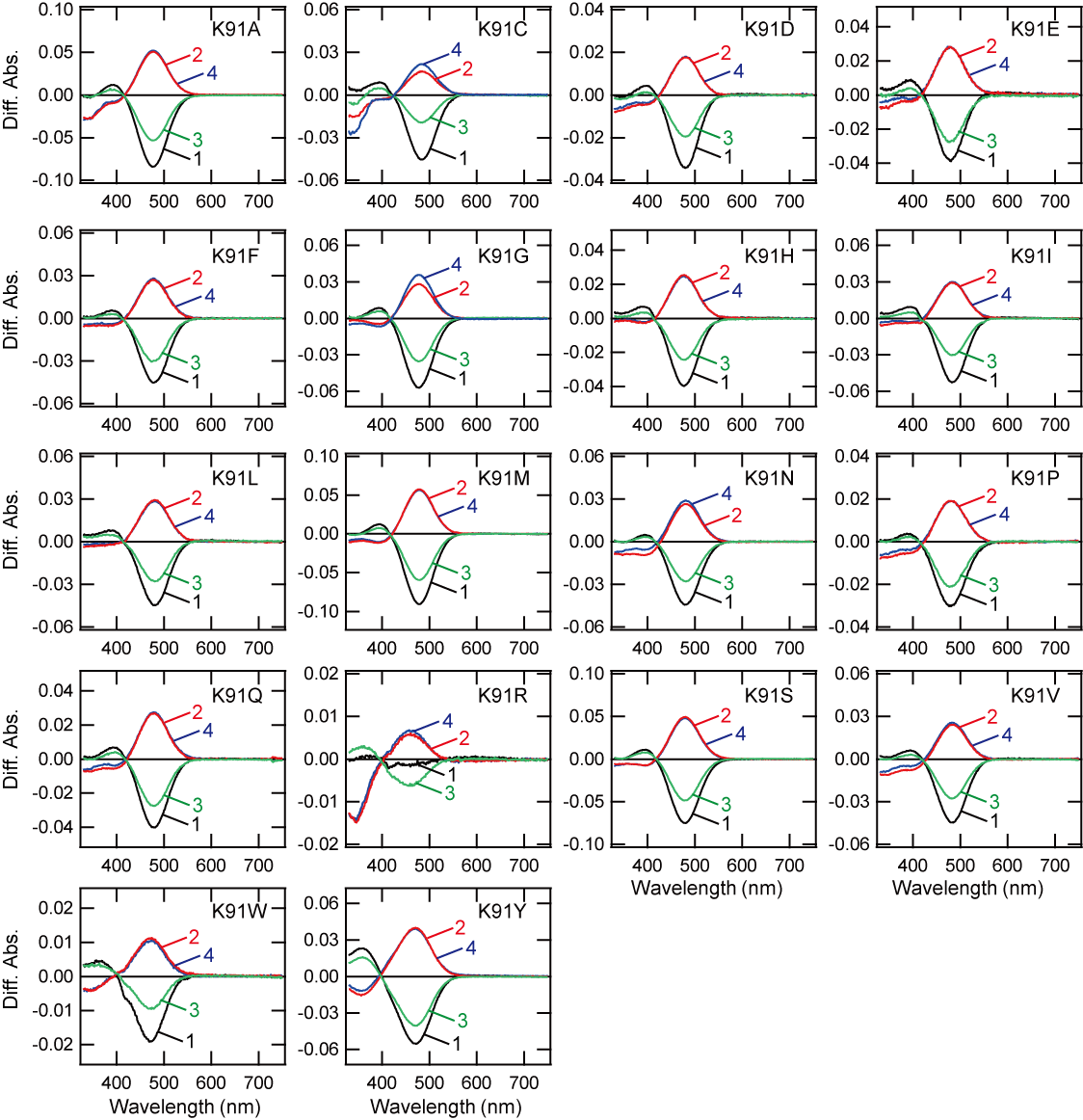
**

**Figure S3 Spectral property of K91 mutants after incubation with all-*trans* retinal**

The cell membranes containing K91 mutants after the addition of all-*trans* retinal were solubilized with 1 % DDM and their absorption spectra were recorded in the dark and after light irradiation. Spectral changes caused by yellow light (>500 nm) irradiation (curve 1), subsequent UV light (360 nm) irradiation (curve 2), yellow light re-irradiation (curve 3) and UV light re-irradiation (curve 4) are shown.

**
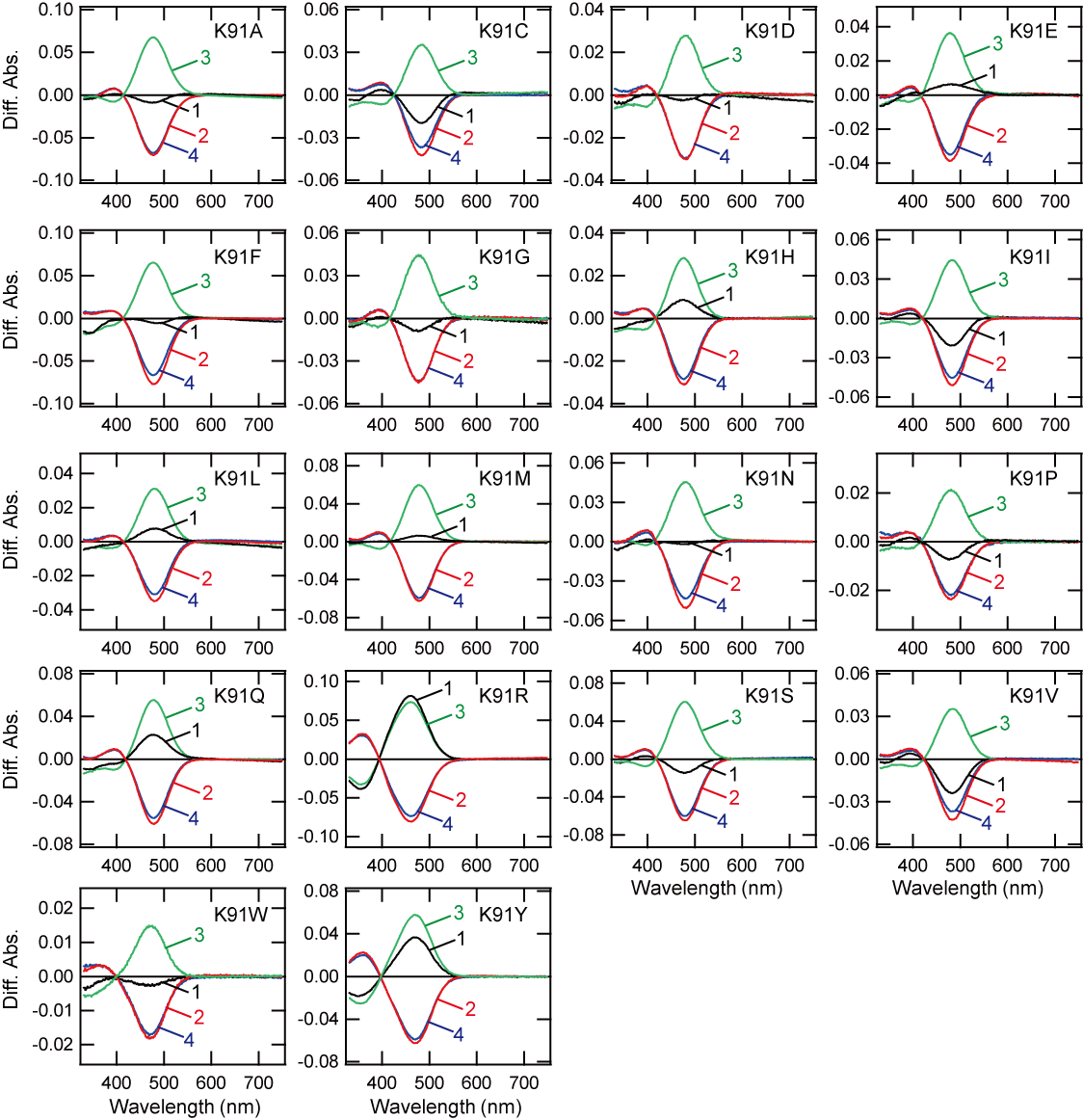
**

**Figure S4 Spectral property of K91 mutants after incubation with 11-*cis* retinal**

The cell membranes containing K91 mutants after the addition of 11-*cis* retinal were solubilized with 1 % DDM and their absorption spectra were recorded in the dark and after light irradiation. Spectral changes caused by UV light (360 nm) irradiation (curve 1), subsequent yellow light (>500 nm) irradiation (curve 2), UV light re-irradiation (curve 3) and yellow light re-irradiation (curve 4) are shown.

**
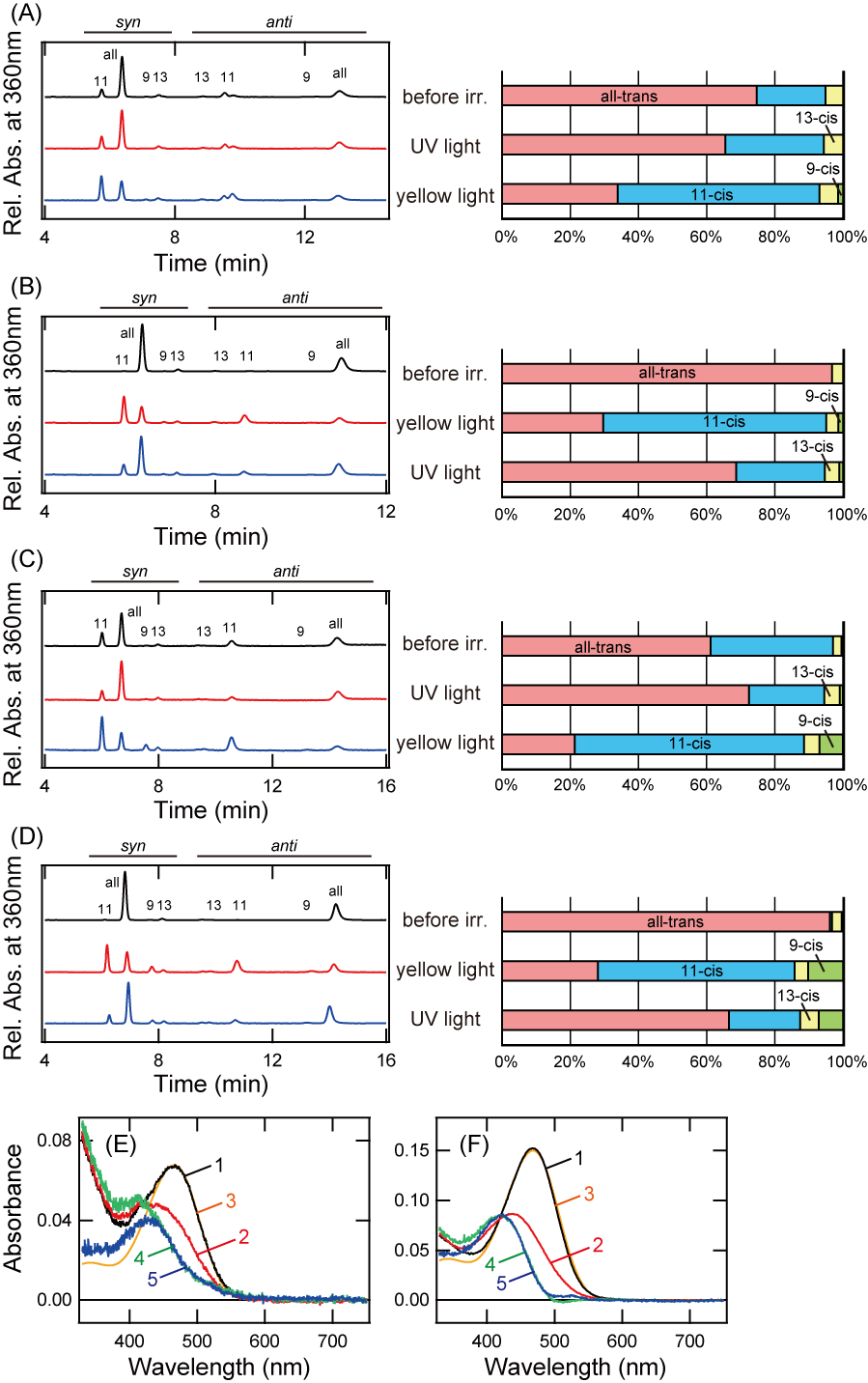
**

**Figure S5 Characterization of K91A and K91Q mutants of Opn5m**

(A, C) Retinal configuration changes of K91A (A) and K91Q (C) mutants purified after the incubation with 11-*cis* retinal. The configurations were analyzed before light irradiation, after UV light (360 nm) irradiation and after subsequent yellow light (>500 nm) irradiation. (B, D) Retinal configuration changes of K91A (B) and K91Q (D) mutants purified after the incubation with all-*trans* retinal. The configurations were analyzed before light irradiation, after yellow light irradiation and after subsequent UV light irradiation. (Left) The retinal configurations were analyzed with HPLC after extraction of the chromophore as retinal oximes (syn and anti forms of 9-*cis*, 11-*cis*, 13-*cis*, and all-*trans* retinal oximes). (Right) Isomeric compositions of retinal before and after light irradiation of the mutant. (E, F) Calculation of absorption spectra of K91A (E) and K91Q (F) mutants. We recorded the absorption spectrum (curve 1) of the samples purified after reconstitution with all-*trans* retinal (the same as curve 1 of Fig. 4B or 4D). This sample contained almost exclusively all-*trans* retinal (see Fig. S5B or S5D). To obtain the absorption spectrum of the 11-*cis* retinal bound form (curve 4), we subtracted curve 1 from curve 2 (the same as curve 2 of Fig. 4B or 4D) based on the component ratio of 11-*cis* and all-*trans* retinals shown in Fig. S5B or S5D. To remove the light scattering component in curves 1 and 4, we fitted curve 1 with a template spectrum modeled by the Lamb and Govardovskii method (1, 2) (curve 3). We also subtracted the difference spectrum between curves 1 and 3 from curve 4 (curve 5). Finally, we normalized curves 3 and 5 to be ~1.0 at λmax of curve 3 to show the normalized spectra in Fig. 4E or 4F.


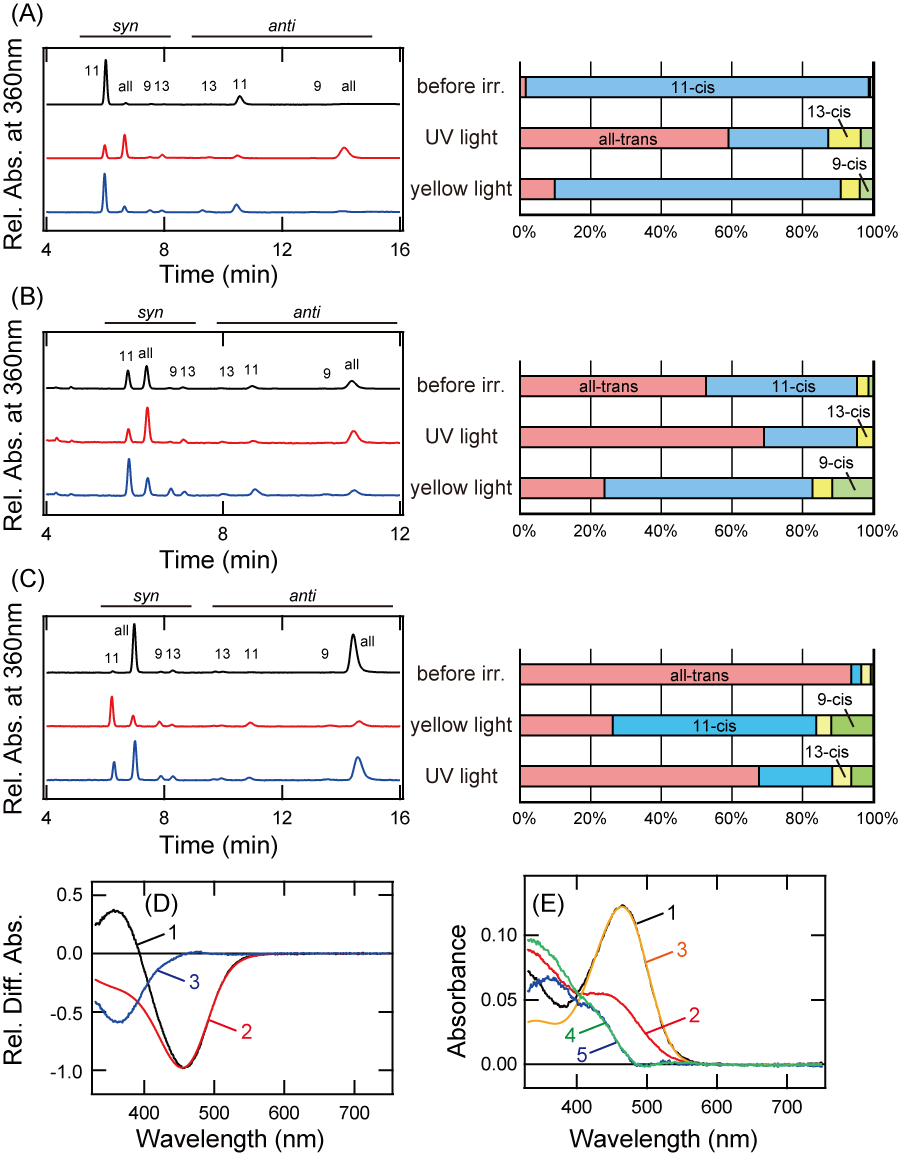


**Figure S6 Characterization of K91R and K91Y mutants of Opn5m**

(A, B) Retinal configuration changes of K91R (A) and K91Y (B) mutants purified after incubation with 11-*cis* retinal. The configurations were analyzed before light irradiation, after UV light (360 nm) irradiation and after subsequent yellow light (>500 nm) irradiation. (C) Retinal configuration changes of K91Y mutant purified after incubation with all-*trans* retinal. The configurations were analyzed before light irradiation, after yellow light irradiation and after subsequent UV light irradiation. (Left) The retinal configurations were analyzed with HPLC after extraction of the chromophore as retinal oximes (syn and anti forms of 9-*cis*, 11-*cis*, 13-*cis*, and all-*trans* retinal oximes). (Right) Isomeric compositions of retinal before and after light irradiation of the mutant. (D) Calculation of absorption spectra of K91R mutant. We normalized the difference spectrum (curve 2 in the inset of Fig. 5A) to be ~ -1.0 at the negative maximum (curve 1). To obtain the spectrum of the all-*trans* retinal bound form (curve 2), we fitted curve 1 with a template spectrum modeled by the Lamb and Govardovskii method (1, 2). To obtain the spectrum of the 11-*cis* retinal bound form (curve 3), we subtracted curve 1 from curve 2. Finally, we normalized curves 2 and 3 to be ~1.0 at the negative maximum of curve 2 to show the normalized spectra in Fig. 5E. (E) Calculation of absorption spectra of K91Y mutant. We recorded the absorption spectrum (curve 1) of the sample purified after reconstitution with all-*trans* retinal (the same as curve 1 of Fig. 5D). This sample almost exclusively contained all-*trans* retinal (see Fig. S6C). To obtain the absorption spectrum of the 11-*cis* retinal bound form (curve 4), we subtracted curve 1 from curve 2 (the same as curve 2 of Fig. 5D) based on the component ratio of 11-*cis* and all-*trans* retinals shown in Fig. S6C. To remove the light scattering component in curves 1 and 4, we fitted curve 1 with a template spectrum modeled by the Lamb and Govardovskii method (1, 2) (curve 3). We also subtracted the difference spectrum between curves 1 and 3 from curve 4 (curve 5). Finally, we normalized curves 3 and 5 to be ~1.0 at λmax of curve 3 to show the normalized spectra in Fig. 5F.


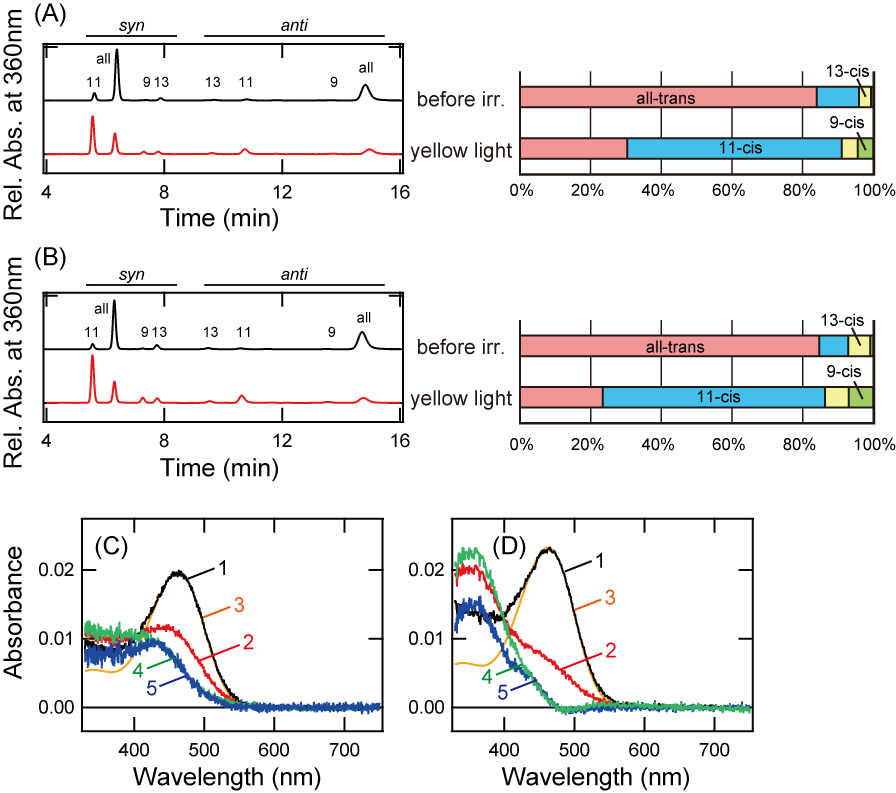


**Figure S7 Characterization of K91Y mutants under pH 6 and 7**

(A, B) Retinal configuration changes of K91Y mutant purified after the incubation with all-*trans* retinal. The configurations were analyzed before light irradiation and after subsequent yellow light (>500 nm) irradiation under pH 6 (A) and pH 7 (B). (Left) The retinal configurations were analyzed with HPLC after extraction of the chromophore as retinal oximes (syn and anti forms of 9-*cis*, 11-*cis*, 13-*cis*, and all-*trans* retinal oximes). (Right) Isomeric compositions of retinal before and after light irradiation of the mutant. (C, D) Calculation of absorption spectra of K91Y mutant under pH 6 (C) and pH 7 (D). We recorded the absorption spectrum (curve 1) of the samples purified after reconstitution with all-*trans* retinal (the same as curve 1 of Fig. 6A or 6B). This sample contained almost exclusively all-*trans* retinal (see Fig. S7A or S7B). To obtain the absorption spectrum of the 11-*cis* retinal bound form (curve 4), we subtracted curve 1 from curve 2 (the same as curve 2 of Fig. 6A or 6B) based on the component ratio of 11-*cis* and all-*trans* retinals shown in Fig. S7A or S7B. To remove the light scattering component in curves 1 and 4, we fitted curve 1 with a template spectrum modeled by the Lamb and Govardovskii method (1, 2) (curve 3). We also subtracted the difference spectrum between curves 1 and 3 from curve 4 (curve 5). Finally, we normalized curves 3 and 5 to be ~1.0 at λmax of curve 3 to show the normalized spectra in Fig. 6C or 6D.

**
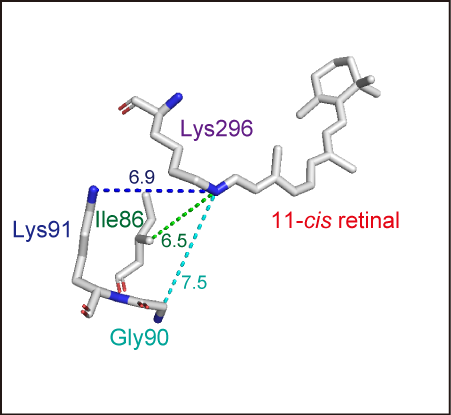
**

**Figure S8 Predicted structural model of the residues at positions 86, 90 and 91 of human Opn5**

Structural model of human Opn5 was constructed based on the crystal structure of bovine rhodopsin (PDB: 1U19) by homology modeling using MOE software (Chemical Computing Group Inc.) and was visualized using PyMOL. The distances between the Schiff base and the residues at positions 86, 90 and 91 are shown.

**Table S1**

λmax of the 11-*cis* retinal and all-*trans* retinal bound forms and the affinity for the retinal isomers in Opn5m K91 mutants

WT K91R K91Y K91A K91Q K91T

λmax of 11-*cis* retinal bound form (nm) 360 362^a^ 360/430 428 424 435

λmax of all-*trans* retinal bound form (nm) 474 454 464 466 468 472

affinity for 11-*cis* retinal +++ +++^b^ ++ + ++ -

affinity for all-*trans* retinal +++ - +++ +++ +++ +++

^a^λmax of the 11-*cis* retinal and all-*trans* retinal bound forms refer to the data shown in Figs. 2, 3, 4 and 5.

^b^Affinity for the retinal isomers is estimated by the comparison among the data shown in Figs. S2, S5 and S6.

**References**

1. Lamb, T. D. (1995) Photoreceptor spectral sensitivities: common shape in the long-wavelength region *Vision Res* **35**, 3083-3091

2. Govardovskii, V. I., Fyhrquist, N., Reuter, T., Kuzmin, D. G., and Donner, K. (2000) In search of the visual pigment template *Vis Neurosci* **17**, 509-528
